# Prosocialness and Happiness in Chilean Student Teachers

**DOI:** 10.3389/fpsyg.2021.745163

**Published:** 2021-11-05

**Authors:** Manuel Mieres-Chacaltana, Sonia Salvo-Garrido, Marianela Denegri

**Affiliations:** ^1^Departamento de Educación Media, Universidad Católica de Temuco, Temuco, Chile; ^2^Programa de Doctorado en Ciencias Sociales, Universidad de La Frontera, Temuco, Chile; ^3^Departamento de Matemática y Estadística, Laboratorio de Investigación en Ciencias Sociales Aplicadas (LICSA), Universidad de La Frontera, Temuco, Chile; ^4^Núcleo Científico y Tecnológico en Ciencias Sociales, Universidad de La Frontera, Temuco, Chile

**Keywords:** prosocialness, happiness, education, teaching, initial teacher training

## Abstract

The aim of the study was to evaluate the relation between prosocialness and happiness in a sample of student teachers (*N*=224, age=21.42). Adapted versions of a prosocialness scale and another on happiness were used. A structural equations model was estimated that presented a suitable fit (CFI=0.951; TLI=0.944 and RMSEA=0.065). The results yielded a positive relation between prosocialness and happiness. Its implications for initial teacher training are discussed.

## Introduction

Prosocialness is defined as a voluntary behavior meant to benefit someone else (Dovidio et al., 2006; Caprara and Steca, 2007; Eisenberg et al., 2015). These benefits can be on both the physical and emotional levels (Catalano et al., 2004; Caprara et al., 2005; Benson et al., 2006). Its study has become important in the last two decades given that these behaviors are desirable and beneficial to society (Eisenberg et al., 2006). It has been stated that prosocial behaviors affect the increase in happiness and well-being (Lyubomirsky, 2001; Lyubomirsky and Layous, 2013).

Happiness has been approached from three psychological perspectives: hedonic, eudaimonic, and flourishing. The first, reduces it to the pursuit of pleasure and the avoidance of pain (Waterman, 2008; Goodman et al., 2018). In contrast, the eudaimonic approach puts the focus on self-actualization and the actualization of human potential (Ryan and Deci, 2001). Consequently, it conceptualizes happiness as a state of fullness of personal functioning and valuing the meaning of life (Ryan and Deci, 2001; Waterman, 2008). Research has also developed complementary propositions (Keyes et al., 2002). Flourishing is one of the designations used to identify this integrated perspective (Seligman, 2011). In the context of the present research, happiness is defined from a broad orientation, encompassing elements of these three streams. That is, as the experience of joy, satisfaction, positive well-being, combined with the feeling that our life is good, meaningful, and worth living (Lyubomirsky, 2008).

At societal level, prosocialness promotes civic virtue, which in turn is positively associated with happiness (Simon and Mobekk, 2019; Lubian, 2020); moreover, it constitutes an effective way to increase happiness sustainably (Lai et al., 2020), which can also involve the economic dimension (Aknin et al., 2020; Dunn et al., 2020). This is relevant since a common goal of human beings is to achieve happiness (Buss, 2000). It should be emphasized that happy people are more successful on different vital planes (Lyubomirsky et al., 2005a): They adapt better to everyday situations and are more resilient to negative experiences (Abbe et al., 2003; Lyubomirsky et al., 2005b; Nielsen and Christensen, 2021).

Based on the previous research, Unanue et al. (2021) studied the relationship between prosociality and happiness applied to the organizational environment. Using a longitudinal model, they considered the concepts of hedonic, eudaimonic, and flourishing happiness and their relationship with prosocial behaviors at work. These proved to be positive predictors of the three types of happiness. In turn, the three types of happiness positively predicted such prosocial behaviors. This model suggests the existence of a virtuous circle of prosociality and well-being in the workplace.

In its relation to education, several studies have agreed on the positive or advantageous aspects of prosocialness. During childhood, for example, it is associated with the positive self-concept (Garaigordobil and García De Galdeano, 2006) and with a better psychosocial adjustment in children and adolescents (Eisenberg and Fabes, 1998; Xiong et al., 2021), as well as with a superior display of social skills and group integration (Plazas et al., 2010) and greater academic achievements by preventing depression and transgressive behavior (Bandura et al., 1996; Cappella et al., 2013; Flórez-Donado et al., 2018; Deng et al., 2021). More concretely, the promotion of prosocialness at school strengthens civic bonds (Luengo et al., 2014) and a better school climate (Luengo et al., 2017). Therefore, it promotes safety, healthy relationships, and the efforts for scholastic improvement (Cohen et al., 2009; Thapa et al., 2013). Consequently, it is to be expected that levels of happiness and well-being will improve in all the actors involved. This is because the cognitive and emotional advantages derived from prosocialness radiate especially to those who are closer to prosocial people (Chancellor et al., 2018). For others, both children and adolescent students, the existing positive relation between prosocial behaviors and self-assessed happiness has been stated (Krettenauer et al., 2019). This relation appears to be positively associated with strength of character, which contributes to coping better with harassment and intimidation in schools (García-Vázquez et al., 2020).

Given the theoretical and empirical background, this study sought to evaluate the relation between prosocialness and happiness in a sample of student teachers. The purpose was to generate knowledge that serves as input to orient future processes of initial teacher training in relation to the dimensions studied. Limiting the issue to the one described also accommodates the recommendations to deepen the study of prosocialness in specific milieus and to address the problem of its measurement (Auné et al., 2014), in particular in adulthood (Caprara et al., 2005). In this respect, it should be added that 86% of Chilean university students – including student teachers – are aged between 18 and 29years (Ministerio de Educación de Chile, 2017). This age range corresponds to so-called emerging adulthood, a transitional stage to adult life. This is characterized as being culturally constructed due to the search for identity and the consolidation of traits that will accompany the individual into adulthood (Barrera and Vinet, 2017); thus, the attitudinal, formative, and value-based trajectory to which the young people are exposed is important.

## Materials and Methods

### Participants

The sample was comprised of 224 student teachers belonging to a university located in the Region of La Araucanía, Chile. 61.6% were women and 38.4% were men. The average age of the participants was 21.42years with a standard (SD) of 2.48years.

### Methodological Approach

The methodological approach was correlational quantitative with a nonexperimental and cross-sectional design (Toro and Parra, 2010). A covariance structure model of interdependence was evaluated, since a correlation among the latent constructs that were studied was proposed (Lévy and Varela, 2006). Consequently, a confirmatory factor analysis (CFA) was applied because the factor loads or saturations were defined in advance (Lévy and Varela, 2006).

### Instruments

Prosocialness was measured with an adapted version of the prosocialness scale for adults by Caprara et al. (2005). This was adjusted and validated in a previous study with Chilean student teachers (*N*=859; age=20.72). It is a one-dimensional model of 13 items (see Table 1) with adequate psychometric properties for use on populations like the one under study. Each item is linked to five categories on an ordinal scale that goes from never (1) to always (5). The items include actions referring to helping (e.g., “I try to help others”); to sharing (e.g., “I easily lend money or other things”); to caring (e.g., “I try to be close and care for those who need it”); and to feeling empathy (e.g., “I easily put myself in the shoes of those who are in an awkward situation”). The internal consistency study applied to the instrument in Chile presented a Cronbach’s alpha=0.867, lower than that obtained by the authors of the original scale in Italy (0.91); however, it also reflected suitable reliability.

**Table 1:** Prosocialness scale for adults by Caprara et al. (2005) (adapted version).

1.	I share my things with my friends.
2.	I try to help others.
3.	I am available for volunteer activities to help those in need.
4.	I help those in need right away.
5.	I do what I can to help others avoid getting into trouble.
6.	I feel what others feel intensely.
7.	I am willing to make my knowledge and skills available to others.
8.	I try to console those who are sad.
9.	I easily lend money and other things.
10.	I easily put myself in the shoes of those who are in an awkward situation.
11.	I try to be close and care for those who need it.
12.	I spend time with those friends who feel lonely.
13.	I immediately feel it when my friends are uncomfortable, even when they do not communicate it to me directly.

Happiness was measured with the subjective happiness scale proposed by Lyubomirsky and Lepper (1999). The version translated and evaluated in Chile by Vera-Villarroel et al. (2011) was used. It is a global measurement of subjective happiness. It focuses on the evaluation of a molar category of well-being in terms of an integral psychological phenomenon. It evaluates happiness from the respondent’s point of view because it supposes that each subject has their own idea of happiness; moreover, they are able to discern if they are happy or not and report it (Lyubomirsky, 2008). It is an instrument comprised of four items (see Table 2), each linked to a seven-point Likert-type scale. The ends range from “not a very happy person” to “a very happy person” (item 1); “less happy” to “more happy” (item 2); and “not at all” to “a great deal” (items 3 and 4). The internal consistency study applied to the instrument in Chile showed a Cronbach’s alpha=0.78 (Vera-Villarroel et al., 2011).

**Table 2:** Subjective happiness scale by Lyubomirsky and Lepper (1999), translated and evaluated by Vera-Villarroel et al. (2011).

1.	In general, I consider myself:
2.	Compared with most of my peers, I consider myself:
3.	Some people are generally very happy. They enjoy life regardless of what is going on, getting the most out of everything. To what extent does this characterization describe you?
4.	Some people are generally not very happy. Although they are not depressed, they never seem as happy as they might be. To what extent does this characterization describe you?

### Procedure

Once the sample had been defined, contact was made with the directors and teachers in the programs the participants were enrolled in. The aims of the study were explained to them and their permission was sought to enter the classrooms and apply the instruments. Another option taken was to contact the students directly in other organizational units. Participation in the study was voluntary and anonymous and all the participants signed an informed consent. In this letter, the objectives and scopes of the investigation were explained. In addition, the confidentiality of the data was guaranteed. It should be noted that anonymity, along with protecting the identity of each participant, helps mitigate the effect of social desirability (Fisher, 1993). The surveys were applied in August and September 2019.

### Data Analysis

A CFA was carried out using polychoric correlation matrix and the mean- and variance-adjusted unweighted least squares method. This method is recommended to analyze ordinal variables with a limited number of categories (Finney and Di Stefano, 2006; Forero et al., 2009).

The reliability indicators applied were Cronbach’s alpha (1951) and the omega coefficient (McDonald, 1999). The latter was used to complement the former because Cronbach’s alpha sees its reliability reduced when applied to ordinal variables (Elosua and Zumbo, 2008). It is also affected by sampling error (Ledesma, 2004). For this, Ventura-León and Caycho-Rodríguez (2017) propose complementing the measurement with the omega coefficient which, in contrast to the alpha coefficient, works with the factor loads and achieves more stable calculations (Gerbing and Anderson, 1988). In this context, omega coefficient values between 0.70 and 0.90 are considered acceptable (Campo-Arias and Oviedo, 2008).

To evaluate the goodness-of-fit of the model, the following indicators were used as: the comparative fit index (CFI), the Tucker-Lewis index (TLI), and the root mean-square error of approximation (RMSEA). From an interpretative perspective, the model presents a suitable fit when the CFI and the TLI display values over 0.90 (Schumacher and Lomax, 1996), whereas for the RMSEA values below 0.08 are considered adequate (Browne and Cudeck, 1993; Gouveia et al., 2018). The analyses were generated with the support of the Mplus program 7.11 (Muthén and Muthén, 2012).

## Results

The proposed model presented a good fit to the data, yielding the following values: CFI=0.951; TLI=0.944 and RMSEA=0.065 (CI90%=0.053 0.078).

The internal consistency indicators produced by the CFA for the prosocialness and subjective happiness scales were as: Cronbach’s alpha=0.843 and 0.756; omega coefficient=0.858 and 0.839, respectively.

**Figure 1:**
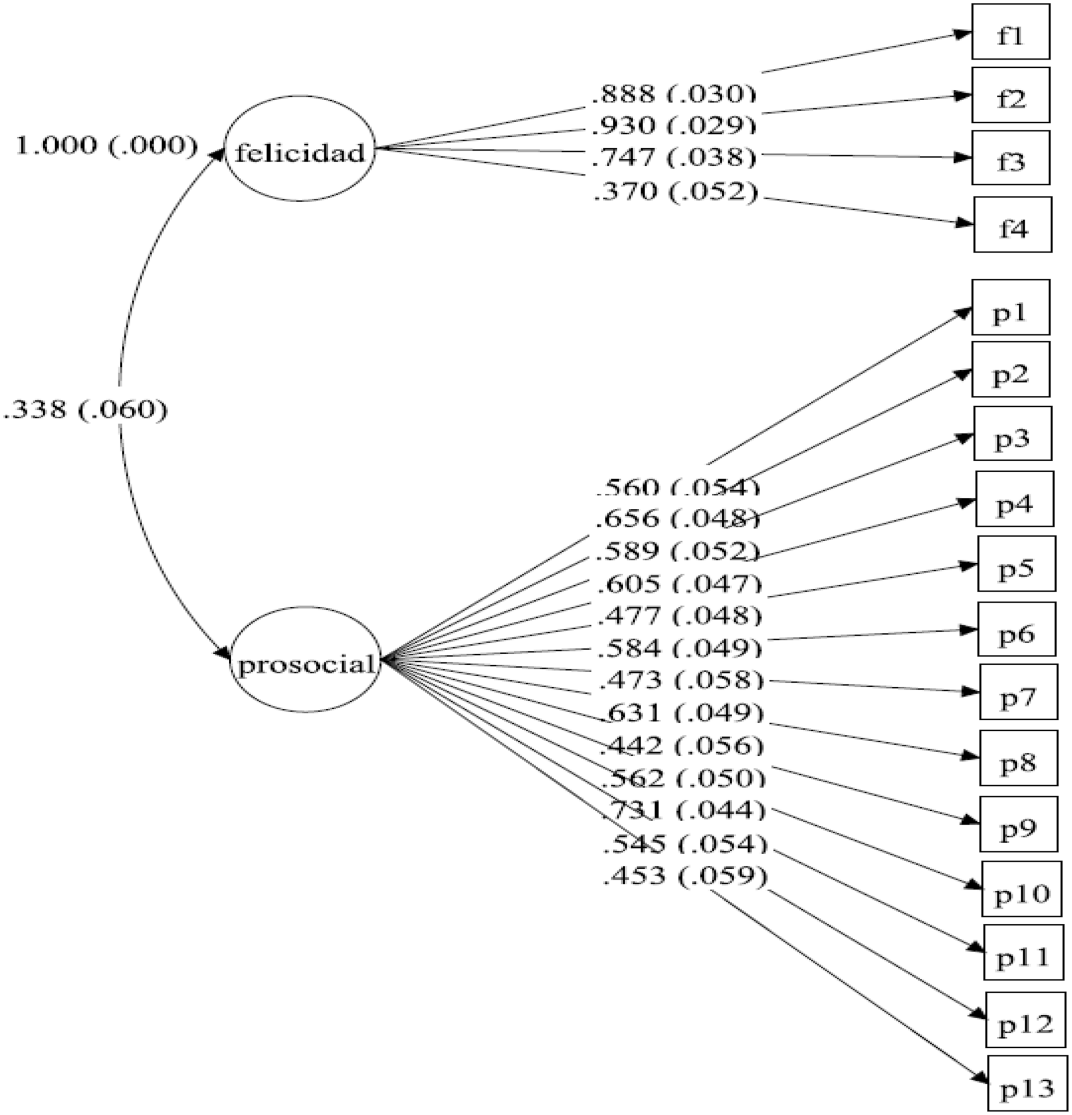
Standardized results of the CFA applied to the model created by the authors with the support of Mplus 7.11 (Muthén and Muthén, 2012).

Figure 1 summarizes the model, including the measurement variables with their factor loads and standard errors. The factor loads varied between 0.442 and 0.731 for prosocialness, and between 0.370 and 0.930 for subjective happiness, all statistically significant (*p*<0.0001). The correlation between the prosocialness and subjective happiness scales was 0.338 and statistically significant (*p*<0.0001).

The results yielded by the CFA applied confirmed a positive relation between prosocialness and happiness. In other words, the standards of prosocial behavior (Dovidio et al., 2006; Caprara and Steca, 2007; Eisenberg et al., 2015) reported by the participants, including its physical and emotional facets (Catalano et al., 2004; Caprara et al., 2005; Benson et al., 2006), were associated positively with their subjective happiness (Lyubomirsky and Lepper, 1999; Vera-Villarroel et al., 2011).

The instruments applied presented suitable levels of reliability. In the case of the adapted version of the prosocialness scale for adults by Caprara et al. (2005), the Cronbach’s alpha, although lower than the one in the original study, was acceptable (Cronbach, 1951). In addition, the value produced by the omega coefficient (McDonald, 1999), applied according to the directions given by Ventura-León and Caycho-Rodríguez (2017), is also admissible (Campo-Arias and Oviedo, 2008). It also provides the results with greater stability (Gerbing and Anderson, 1988). This is due to the problems presented by the Cronbach’s alpha when applied to ordinal variables (Ledesma, 2004; Elosua and Zumbo, 2008), like those in the present study.

In the same vein, the subjective happiness scale presented a slightly lower Cronbach’s alpha than that of the validation study conducted in Chile (0.756 vs. 0.780). The result of the omega coefficient – the advantages of which have already been explained – served to confirm what was indicated.

In relation to the goodness-of-fit indicators, both the CFI and the TLI were within the ranges that the literature considers acceptable (Schumacher and Lomax, 1996). The same occurred with the RMSEA value (Browne and Cudeck, 1993; Gouveia et al., 2018).

## Discussion

First of all, and on a general level, the positive relation between prosocialness and happiness was consistent with the results of other studies (Lyubomirsky, 2001; Lyubomirsky and Layous, 2013; Unanue et al., 2021). This extends to the economic dimension if it is considered that item 9 on the prosocialness scale for adults specifically refers to this (“I easily lend money or other things”). In this sense, this part of the results is in line with those studies that report a positive connection between prosocial economic behaviors associated with higher happiness levels (Aknin et al., 2020; Dunn et al., 2020). However, more research is needed to make a more definitive statement about this link. What could be truly interesting is if the focus was confined to a university education environment like that of students who have only limited resources. It could also anticipate the response by the subjects about being professionals, since the teaching profession is characterized by mid-level salaries. This is because the recently reviewed theoretical and empirical considerations could be applied to a specific professional area of great potential for the development of prosocial behaviors.

Second, if the discussion is limited to the education sphere, it is worth emphasizing the application of the instruments in a context of initial teacher training; particularly in the case of the prosocialness construct, where the preceding study has recommended the development and/or adaptation of measuring instruments to specific institutional areas (Auné et al., 2014), and in specific age groups, such as adulthood (Caprara et al., 2005), which is also interesting for the discussion, because the study participants can be classified as emerging adults (Barrera and Vinet, 2017). Moreover, their age distribution was consistent with the statistics produced by official entities (Ministerio de Educación de Chile, 2017). This is relevant because it is an evolutionary stage in search of identity and consolidation of characteristics that will accompany them into adult life. More importantly, however, is their future condition as teachers, added to implications that their prosocial standards may have for their later professional behavior; hence, confirming this relation anticipates prosocial behaviors in future professional work, which together with the link to happiness could result in better job satisfaction (Nielsen and Christensen, 2021). This is due to the increasing number of reports that place prosocialness as positively associated with socioemotional development (Baiocco et al., 2018; García and Tully, 2020), which in turn is related to a wide range of issues, such as the development of self-esteem (Garaigordobil and García De Galdeano, 2006), psychosocial adjustment (Eisenberg and Fabes, 1998; Xiong et al., 2021), and group integration (Plazas et al., 2010). Consequently, it is also related to better academic results (Bandura et al., 1996; Cappella et al., 2013; Flórez-Donado et al., 2018), which subsequently reinforces the indications that point out the relevance of socioemotional education in teacher training, which can amplify the effect of positive modeling they would have for their future students (Cefai et al., 2018; García-Vázquez et al., 2020; Zee et al., 2020; Oliveira et al., 2021; Sezen-Gultekin et al., 2021).

Third, the results lead to thinking about the social relevance of school and its potential to make substantive changes in individuals and therefore society. For example, in areas, such as civic education, if it is conceived not only as a cognitive exercise for learning concepts but also is stimulated with prosocial behaviors, its potential contribution in the formation of happier citizens is augmented (Simon and Mobekk, 2019; Lubian, 2020) and from multiple perspectives of happiness (Ryan and Deci, 2001; Keyes et al., 2002; Lyubomirsky, 2008; Waterman, 2008; Seligman, 2011; Goodman et al., 2018). At the same time, they would be more committed to the issues of their community and its development (Eisenberg et al., 2006; Grant and Dutton, 2012; Luengo et al., 2014). And in a reciprocal way, better conditions for the development of each individual would be established. This is in terms of the provision and internalization of resources to confront various life problems (Abbe et al., 2003; Lyubomirsky et al., 2005a,b) and thus to facilitate the construction of several personal and collective projects for happiness and a good life (Buss, 2000). Without a doubt, school is one of the socialization spaces to teach these behaviors and put them into practice, especially if it is considered that the development of social skills, the ability for self-regulation, and self-esteem is important for personal growth. This becomes a valuable resource to tackle complex events like intimidation and harassment (García-Vázquez et al., 2020). In all these processes, the facilitating and modeling role of teachers can be fundamental in terms of their leadership and proximity to the possible beneficiaries of their actions (Chancellor et al., 2018; Deng et al., 2021), especially with respect to their contributions to establishing a suitable school climate and its resulting beneficial effects (Cohen et al., 2009; Thapa et al., 2013; Luengo et al., 2017).

Thus, the behaviors focused on the benefit of others are fundamental to promoting education, safeguarding health, and combatting poverty and hunger (Grant and Dutton, 2012). Several studies recommend introducing social policies that promote the development of prosocial competences and behaviors to increase children’s happiness (Baiocco et al., 2018). At the same time, the recognition of these expressions should be fostered from an early age, since they provide positive opportunities for socialization (García and Tully, 2020). This addresses the processes of initial teacher training directly because students’ socioemotional development requires the social and emotional training of the teachers (Cefai et al., 2018; García-Vázquez et al., 2020; Zee et al., 2020; Oliveira et al., 2021; Sezen-Gultekin et al., 2021).

## Conclusion

This study evaluated the relation between prosocialness and subjective happiness in a sample of student teachers at a university in the Region of La Araucanía, Chile. The proposed model presented suitable levels of reliability and fit.

Although the sample size was acceptable in terms of the number of items considered, the non-probabilistic nature of the study does not permit these results to be generalized to the rest of the population. This trait is the most relevant limitation of this study. It should be added that the searched data were self-reported by the participants with the resulting impact that social desirability and memory bias could have had on the responses. Finally, the homogenizing nature of the scale responses does not account for the inherent specificities that social phenomena have, always located territorially and historically.

However, the evaluated model forms a basic line for the development of future investigations referring to the relation between prosocialness and happiness in the field of teacher training. Given the dynamics that these constructs represent in relation to the evolution of life, longitudinal studies are recommended that incorporate more variables. For example, age (in more specific ranges), gender, ethnic group, and the effects of the curriculum and the teaching specialty, among others. On the last point, the coordination of training proposals referring to the development of socioemotional skills with their corresponding evaluations would be interesting given the increasing number of investigative reports that recommend their inclusion in the teacher training curriculum.

## Data Availability Statement

The original contributions presented in the study are included in the article/supplementary files, further inquiries can be directed to the corresponding author.

## Ethics Statement

Ethical approval was not provided for this study on human participants because it is a preliminary study to the development of a doctoral thesis. The doctoral thesis project was submitted to the Scientific Ethics Committee of the University of La Frontera. The patients/participants provided their written informed consent to participate in this study.

## Author Contributions

MM-C created the research question, conducted a bibliographic search, theoretical framework, methodological design, data analysis, and contributed to the discussion. SS-G contributed the methodological design, data analysis, and discussion. MD contributed to the theoretical framework, methodological design, and discussion. All authors contributed to the article and approved the submitted version.

## Funding

This work was produced within the framework of FONDECYT project no. 1210551.

## Conflict of Interest

The authors declare that the research was conducted in the absence of any commercial or financial relationships that could be construed as a potential conflict of interest.

## Publisher’s Note

All claims expressed in this article are solely those of the authors and do not necessarily represent those of their affiliated organizations, or those of the publisher, the editors and the reviewers. Any product that may be evaluated in this article, or claim that may be made by its manufacturer, is not guaranteed or endorsed by the publisher.
